# Sequential assessment of clinical and laboratory parameters in patients with hemorrhagic fever with renal syndrome

**DOI:** 10.1371/journal.pone.0197661

**Published:** 2018-05-23

**Authors:** Emil Pal, Miša Korva, Katarina Resman Rus, Nataša Kejžar, Petra Bogovič, Anica Kurent, Tatjana Avšič-Županc, Franc Strle

**Affiliations:** 1 Department of Infectious Diseases, Murska Sobota General Hospital, Rakičan, Slovenia; 2 Faculty of Medicine, University of Ljubljana, Ljubljana, Slovenia; 3 Institute of Microbiology and Immunology, Faculty of Medicine, University of Ljubljana, Ljubljana, Slovenia; 4 Institute for Biostatistics and Medical Informatics, Faculty of Medicine, University of Ljubljana, Ljubljana, Slovenia; 5 Department of Infectious Diseases, University Medical Center Ljubljana, Ljubljana, Slovenia; 6 Novo Mesto General Hospital, Department of Infectious Diseases, Novo Mesto, Slovenia; University of Minnesota College of Veterinary Medicine, UNITED STATES

## Abstract

**Background:**

Information on the sequential appearance, duration, and magnitude of clinical and laboratory parameters in hemorrhagic fever with renal syndrome (HFRS) is limited.

**Methods:**

Analysis of clinical and laboratory parameters obtained serially in 81 patients with HFRS, of whom 15 were infected with Dobrava virus and 66 with Puumala virus.

**Results:**

The initial signs/symptoms, appearing on median day 1 of illness, were fever, headache, and myalgia. These were present in 86%, 65%, and 40% of patients and had a median duration of 4, 4, and 5.5 days, respectively. The signs/symptoms were followed by myopia (appearance on day 5), insomnia (day 6), oliguria/anuria (day 6), polyuria (day 9), and sinus bradycardia (day 9.5). These were present in 35%, 30%, 28%, 91%, and 35% of patients; their median duration was 2, 2, 2, 7, and 1 day, respectively. Laboratory abnormalities, including thrombocytopenia, elevated alanine aminotransferase, CRP, procalcitonin, creatinine, diminished glomerular filtration rate, and leukocytosis, were ascertained on admission to hospital or on the following day (day 5 or 6 of illness) and were established in 95%, 87%, 99%, 91%, 94%, 87%, and 55% of patients, and had a median duration of 4, 3, 7, 3, 9, 8, and 2 days, respectively. Comparison of patients infected with Dobrava and Puumala viruses found several differences in the frequency, magnitude, and duration of abnormalities, indicating that Dobrava virus causes the more severe HFRS.

**Conclusions:**

In the majority of patients, the classic clinical distinction into febrile, hypotonic, oliguric, polyuric, and convalescent phases of illness is unclear.

## Introduction

Pathogenic hantaviruses are the etiologic agents of two clinical syndromes in humans, namely hemorrhagic fever with renal syndrome (HFRS) in Eurasia and hantavirus (cardio)pulmonary syndrome in the Americas [1]. The viruses are primarily carried by rodents, shrews, moles, and bats [2]. Transmission to humans occurs via aerosols or dust particles of virus-contaminated rodent urine, feces, or saliva, and probably also via food or hands contaminated by these excretions [3].

The clinical spectrum of hantavirus infections ranges from asymptomatic infection to severe disease with fatal outcome, depending, in part, on the causative virus [4–8]. The onset of HFRS is abrupt with fever accompanied by myalgia, headache, transient myopia, nausea, vomiting, diarrhea, abdominal pain, back pain, flushed face, and dizziness [5, 6, 9]. Although renal injury is a distinctive feature of HFRS, various extrarenal manifestations can develop, with pulmonary, hemorrhagic, pancreatobiliary, central nervous system, endocrine, and cardiovascular events [7, 10, 11].

The pathogenesis of HFRS is only partially understood [12]. Capillary leakage is the characteristic of hantavirus disease, resulting in tissue edema and organ failure [2, 13].

In Slovenia, a small Central European country of approximately 20,000 km^2^ and with two million inhabitants, several small mammal species have been confirmed to harbor hantaviruses: *Apodemus flavicollis* (Dobrava virus, genotype Dobrava, DOBV), *Apodemus agrarius* (Dobrava virus, genotype Kurkino, DOBV-Kurkino), *Myodes glareolus* (Puumala virus, PUUV), *S*. *areanus* (Seewis virus), *Microtus agrestis*, *Microtus arvalis* and *Microtus subterraneus* (Tula virus). Three of the viruses, namely the DOBV, DOBV–Kurkino and PUUV, cause disease in humans, with significant differences in the severity of illness. Disease caused by PUUV and DOBV- Kurkino has usually a mild clinical course with a fatality rate <1%, whereas DOBV infections cause a more severe form of HFRS with an approximate 10% case fatality rate [5, 6, 14–16]. In the 31-year period from 1985 to 2015, 544 cases of HFRS (136 infected with DOBV, 4 infected with DOBV-Kurkino and 404 with PUUV) were identified in Slovenia; 188 of these were diagnosed in 2012, when the largest epidemics of HFRS occurred.

The main aim of our study was to gain detailed insight into HFRS dynamics by analyzing sequential (mostly daily) values of selected clinical and laboratory parameters in patients with HFRS caused by PUUV or DOBV (genotype Dobrava) infection.

## Patients and methods

### Ethics

The study was conducted according to the principles expressed in the Declaration of Helsinki and was approved by the Slovenian National Medical Ethics Committee (69/03/12). Written informed consent was obtained from all the patients.

### Patients

The study comprised patients with HFRS hospitalized in three Slovenian hospitals (General Hospital Murska Sobota, University Medical Center Ljubljana, and General Hospital Novo mesto) in the years 2012–2013, for whom information was obtained prospectively, and a subset of patients participating in previous studies in the years 2007–2011 [8, 15, 17]. All the patients fulfilled three basic criteria: (a) an illness clinically compatible with HFRS and substantiated with at least two of three findings (fever >38°C, acute renal failure, thrombocytopenia); (b) acute hantavirus infection confirmed by immunofluorescence assay [6]; and (c) available serial (at least every other day during a minimum period of 5 days) follow-up of clinical findings and laboratory parameters. The patients were grouped according to the etiology (HFRS caused by PUUV or DOBV), each of the two groups being further classified into subgroups with mild or severe disease. The latter classification was based on clinical and laboratory parameters [8, 15]. Criteria for severe HFRS were: 1. The need for dialysis, or 2. The lowest systolic blood pressure <90 mm Hg and/or clinical signs of shock, or 3. Thrombocytopenia <50 x109/L and the presence of a) bleeding and/or b) renal failure manifested with oliguria (diuresis <500 mL/day) and/or >4 x higher than the upper normal level of urea or creatinine.

### Data collection

Clinical data and laboratory findings were obtained serially (typically daily) from each patient by means of a standardized questionnaire as a part of prospective study in the years 2012–2013 and/or evaluation of medical records for a subset of patients participating in previous studies. Clinical data consisted of fever, systolic blood pressure, diuresis, headache, myalgia, insomnia, heart rate, acute myopia, dizziness, ascites, pleural effusion, and hemorrhagic manifestations. Laboratory findings included leucocyte and platelet counts, concentrations of C-reactive protein (CRP), procalcitonin (PCT) and creatinine, glomerular filtration rate (eGF) estimated using the Cockcroft-Gault equation, alanine aminotransferase (ALT) level, serum albumin concentration, prothrombin time (PT) expressed as the international normalized ratio, and activated partial thromboplastin time (aPTT).

Serum IgM and IgG antibodies against PUUV and DOBV were determined using commercially available enzyme immunoassays: Reagena DOBRAVA-HANTAAN IgM EIA, Reagena DOBRAVA-HANTAAN IgG EIA, Reagena PUUMALA IgM EIA and Reagena PUUMALA IgG EIA (Reagena International Oy Ltd, Toivala, Finland), as described elsewhere [15, 17]. For determination of the hantavirus genotype and daily viral load, total RNA was extracted from whole blood samples using TRIzol™ Reagent (ThermoFisher Scientific) according to the manufacturer’s instructions. Hantavirus RNA was quantified in a multiplex one-step real-time RT-PCR assay specific for Slovenian DOBV and PUUV, as previously described [17].

### Statistical methods

Medians and ranges were used for numerical variables and percentages for categorical variables. The Mann–Whitney and Fisher exact tests were used for comparison of numerical and categorical variables between two groups, respectively. Population values were indicated by 95% confidence intervals for percentages. To control for the false discovery rate, reported *P* values were adjusted using the Benjamini–Hochberg correction procedure. R statistical language was used for the analysis [18].

## Results

Eighty-one out of 155 (53%) HFRS patients treated in three Slovenian hospitals in the period 2007–2013 had available serial clinical findings and laboratory parameters and thus fulfilled the study inclusion criteria. Of these 81 patients, 48 (59%) were enrolled in the study in the years 2012 and 2013 (for them information was obtained prospectively) while 33 participated in previous studies in the years 2007–2011 [8, 15, 17]; the latter represent all patients for whom serial clinical findings and laboratory parameters were available. The median age of the patients was 39 (range 18–81) years; 66 patients (81%) were male. Among the 81 patients, 66 were infected with PUUV and 15 with DOBV, genotype Dobrava. The main assumed route of infection was through inhalation during outdoor activities (e.g., farmers, recreational activities, gardeners, bricklayers) or during cleaning of basements or attics. One quarter of patients recalled direct contact with rodents 2–3 weeks before disease onset. In 41/81 (51%) patients the illness began between June and August. Primary care physicians referred the patients to hospital with various diagnoses, most commonly with unspecified febrile illness, enterocolitis, or acute pyelonephritis; HFRS was the referral diagnosis in only four cases (Table 1). The initial symptoms of illness began 5 (1–12) days before admission to hospital: 5 (1–11) days in patients infected with PUUV and 6 (3–12) days in patients with DOBV. The median hospitalization time was 10 (4–74) days; the corresponding durations of hospitalization for PUUV and DOBV infected patients were 10 (4–32) and 22 (7–74) days, respectively (*P* = .012). Among the 81 patients, 56 (69%) had mild HFRS and 25 (31%) the severe form. In the PUUV group, 16/66 (24%) patients had severe disease; the corresponding finding for the DOBV group was 9/15 (60%) (*P* = .056). One patient in the DOBV group died.

**Table 1:** Diagnoses* with which patients were referred to hospital by primary care physicians.

	Patients infected with PUUV (No. = 66)	Patients infected with DOBV (No. = 15)
Unspecified febrile illness	27 (41%)	5 (33%)
Enterocolitis	8 (12%)	3 (20%)
Acute pyelonephritis	4 (6%)	3 (20%)
Meningitis	3 (5%)	0
HFRS	3 (5%)	1 (7%)
Sepsis	3 (5%)	1 (7%)
Pancreatitis	2 (3%)	1 (7%)
Ehrlichiosis	2 (3%)	0
Viral infection	1 (1%)	2 (13%)
Other referral diagnosis**	9 (14%)	0

DOBV Dobrava virus; PUUV Puumala virus

*Some patients had more than one referral diagnosis.

**Diagnoses that were given to individual patients with HFRS included acute kidney failure, acute respiratory failure, blurred vision, headache, pneumonia, vomiting, hepatopathia, endocarditis, and thrombocytopenia.

**Fig 1:**
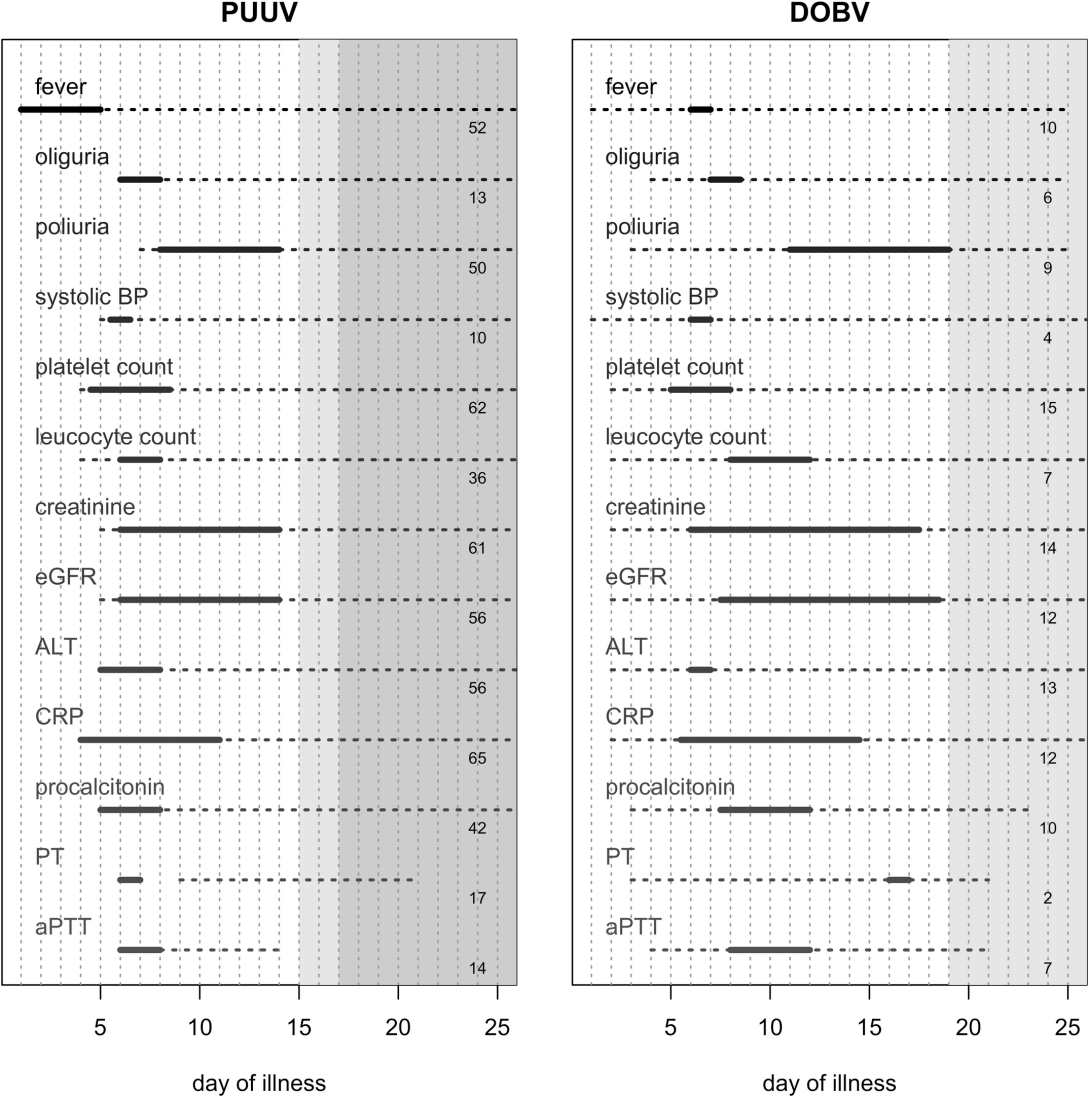
Clinical and laboratory parameters in patients with hemorrhagic fever with renal syndrome infected with Puumala or Dobrava virus. The number of patients with measured individual parameters is shown on the right-hand edge of the picture. The length of the solid line shows the median of symptom duration; the start of the line equates to the median day of symptom onset (see Tables 2 and 3). The dashed line indicates days of illness where at least four measurements were obtained (dashed lines for diuresis in patients with Dobrava virus start only at day 6 of illness; PT and aPTT measurements have a relatively small number of patients with sufficient measurements). Light shaded area corresponds to days of illness when up to 50% of patients were still hospitalized. Dark shaded area corresponds to days of illness when up to 33% of patients were still hospitalized. Definitions: fever: ≥38.0°C; oliguria: ≤500 mL/day; polyuria: ≥2500 mL/day; systolic blood pressure (BP): ≤90 mmHg; platelet count: <130 x 10^9^/L; leukocytosis: >10 x 10^9^/L; elevated creatinine: >97 μmol/L; diminished eGFR: <60 mL/min/1.73 m^2^; elevated serum ALT: >0.56 μkat/L; elevated CRP: >5 mg/L; elevated procalcitonin (PCT): >0.5 μg/L; prolonged prothrombin time (PT): >1, activated partial thromboplastin time (PTT): >36 s. PUUV, Puumala virus; DOBV, Dobrava virus.

Results of serial (typically daily) measurements of clinical and laboratory parameters in patients with HFRS caused by DOBV/PUUV are shown in Tables 2 and 3, and outlined in Fig 1 and S1 Fig. Detailed information on their dynamics is given in S2 and S3 Figs. In the majority of patients the classic clinical distinction into febrile, hypotonic, oliguric, polyuric, and convalescent phases of illness was not evident.

**Table 2:** Serial clinical findings in patients with Hemorrhagic Fever with Renal Syndrome (HFRS).

Clinical findings	All patients with HFRS No. = 81				HFRS caused by PUUV No. = 66				HFRS caused by DOBV No. = 15				*P* value	Adjusted *P* value
	Proportion with symptom/sign	Onset		Duration (days)	Proportion with symptom/sign	Onset		Duration (days)	Proportion with symptom/sign	Onset		Duration (days)		
		day of illness	*before admission : at admission (%)			day of illness	*before admission : at admission (%)			day of illness	*before admission : at admission (%)			
Fever (≥38.0°C)	62/72 (86; 75–93)	1 (1–21)	74 : 15	4 (1–9)	52/60 (87; 75–94)	1 (1–8)	83 : 17	4 (1–9)	10/12 (83; 51–97)	6 (1–21)	30 : 0	1 (1–5)	0.003	0.104
Headache	53/81 (65; 54–75)	1 (1–14)	66 : 21	4 (1–18)	47/66 (71; 59–81)	1 (1–14)	70 : 19	4 (1–18)	6/15 (40; 17–67)	6 (1–8)	33 : 33	1 (1–11)	0.034	0.105
Myalgia	32/81 (40; 29–51)	1 (1–6)	72 : 25	5.5 (2–16)	30/66 (45; 33–58)	1 (1–6)	70 : 27	5 (2–16)	2/15 (13; 2–42)	2 (1–3)	100 : 0	8 (8–8)	0.224	0.397
Dizziness†	13/81 (16; 9–26)	1 (1–12)	69 : 23	4 (1–17)	11/66 (17; 9–28)	1 (1–12)	73 : 18	3 (1–17)	2/15 (13; 2–42)	4.5 (4–5)	50 : 50	6.5 (6–7)	1	1
Myopia	28/81 (35; 25–46)	5 (2–12)	36 : 25	2 (1–33)	24/66 (36; 25–49)	5 (2–12)	38 : 29	2 (1–33)	4/15 (27; 9–55)	6 (3–7)	25 : 0	3 (1–6)	0.560	0.739
Insomnia	24/81 (30; 20–41)	6 (1–35)	25 : 21	2 (1–15)	19/66 (29; 19–41)	6 (1–15)	26 : 16	2 (1–15)	5/15 (33; 13–61)	8 (1–35)	20 : 40	4 (1–5)	0.759	0.886
Systolic blood pressure (≤90 mmHg)†	14/73 (19; 11–30)	6 (2–27)	7 : 36	1 (1–2)	10/61 (16; 9–29)	5.5 (2–8)	10 : 40	1 (1–2)	4/12 (33; 11–65)	6 (3–27)	0 : 25	1 (1–1)	0.227	0.397
Oliguria (≤500 mL/day)	18/65 (28; 18–40)	6 (4–10)	0 : 21	2 (1–12)	12/56 (21; 12–35)	6 (4–9)	0 : 31	2 (1–3)	6/9 (67; 31–91)	7 (6–10)	0 : 0	1.5 (1–12)	0.011	0.056
Polyuria (≥2500 mL/day)	59/65 (91; 80–96)	9 (3–21)	0 : 3	7 (1–37)	50/56 (89; 77–96)	8 (3–13)	0 : 4	6 (1–16)	9/9 (100; 63–100)	11 (6–21)	0 : 0	8 (5–37)	0.002	**0.015**
Bradycardia	28/81 (35; 25–46)	9.5 (4–26)	0 : 0	1 (1–10)	24/66 (36; 25–49)	9.5 (4–14)	0 : 0	1 (1–7)	4/15 (27; 9–55)	9 (6–26)	0 : 0	3 (1–10)	0.560	0.739
Ascites†	11/81 (14; 7–23)	7 (3–53)	9 : 27	1 (1–10)	6/66 (9; 4–19)	7.5 (5–8)	0 : 17	1 (1–1)	5/15 (33; 13–61)	7 (3–53)	20 : 40	7 (1–10)	0.027	0.104
Minor alveolar pulmonary infiltrates†	4/81 (5; 2–13)	7.5 (5–23)	0 : 50	1 (1–8)	2/66 (3; 1–11)	5 (5–5)	0 : 50	1 (1–1)	2/15 (13; 2–41)	15.5 (8–23)	0 : 50	4.5 (1–8)	0.154	0.332
Pleural effusions†	12/81 (15; 8–25)	8 (3–37)	0 : 25	1.5 (1–14)	5/66 (8; 3–18)	8 (5–10)	0 : 20	1 (1–10)	7/15 (47; 22–73)	8 (3–37)	0 : 29	7 (1–14)	<0.001	**0.012**
Bleeding, major **†	7/81 (9; 4–18)	7 (2–38)	14 : 29	4 (1–23)	4/66 (6; 2–16)	6 (2–12)	25 : 25	2.5 (1–4)	3/15 (20; 5–49)	8 (4–38)	0 : 33	5 (3–23)	0.114	0.267
Bleeding, minor ***†	2/81 (2; 0–9)	4.5 (3–6)	0 : 50	2 (1–3)	1/66 (2; 0–9)	3 (3–3)	0 : 100	3 (3–3)	1/15 (7; 0–34)	6 (6–6)	0 : 0	1 (1–1)	0.338	0.530

The order of shown signs/symptoms present in >15% of patients is according to the day of onset in the course of illness.

Data are median (range) or frequencies (percentage; 95% confidence interval).

(Adjusted) *P* values refer to the difference between PUUV and DOBV groups in "Proportion with symptom/sign".

Onset and duration of symptom (Me; Min–Max) are calculated only for patients with the symptom/sign.

Duration is defined as the number of subsequent days with symptom from its onset. Not all laboratory measurements were taken each day, therefore the medical expert (EP) imputed unambiguous days with symptoms for the hospitalized patients.

All measurements from patients are taken into account (including measurements after day 25 of illness).

* Proportion (%) of patients with the onset of individual symptom/sign.

**Bleeding from gastrointestinal, genitourinay and pulmonary sites.

*** Petechiae, ecchymosis, epistaxis without major bleeding.

†The statistical power is low because of the small number of patients with a given parameter.

**Table 3:** Laboratory findings in patients with Hemorrhagic Fever with Renal Syndrome (HFRS).

Laboratory findings	All patients with HFRS No. = 81					HFRS caused by PUUV No. = 66					HFRS caused by DOBV No. = 15					*P* value	Adjusted *P* value
	Proportion of patients with abnormal laboratory test results	Onset		The most abnormal value	Duration (days)	Proportion of patients with abnormal laboratory test results	Onset		The most abnormal value	Duration (days)	Proportion of patients with abnormal laboratory test results	Onset		The most abnormal value	Duration (days)		
		day of illness	*before admission: at admission (%)				day of illness	*before admission: at admission (%)				day of illness	*before admission: at admission (%)				
Platelets <130 x 10^9^/L	77/81 (95; 87–98)	5 (1–12)	16: 71	55 (5–128)	4 (1–16)	62/66 (94; 84–98)	4.5 (1–11)	15: 76	60.5 (20–128)	4 (1–16)	15/15 (100; 75–100)	5 (3–12)	20: 53	38 (5–113)	3 (1–11)	1	1
Leukocytes >10 x 10^9^/L	43/78 (55; 43–66)	6 (2–58)	2: 49	12.6 (10.1–34.7)	2 (1–17)	36/66 (55; 42–67)	6 (2–20)	3: 53	12.5 (10.1–31.8)	2 (1–17)	7/12 (58; 29–84)	8 (4–58)	0: 29	18.3 (12.6–34.7)	4 (1–9)	1	1
Creatinine >97 μmol/L	75/80 (94; 85–98)	6 (1–12)	8: 61	329 (98–1023)	9 (1–72)	61/66 (92; 82–97)	6 (1–11)	8: 64	259 (98–1023)	8 (1–24)	14/14 (100; 73–100)	6 (3–12)	7: 50	654.5 (324–975)	11.5 (1–72)	0.580	0.739
eGFR <60 mL/min/1.73 m^2^	67/77 (87; 77–93)	6 (2–12)	6: 49	18 (5–56)	8 (1–72)	56/66 (85; 73–92)	6 (2–11)	5: 48	20 (5–56)	8 (1–21)	11/11 (100; 68–100)	7.5 (3–12)	8: 50	10 (5–19)	11 (2–72)	0.341	0.530
Serum ALT >0.56 μkat/L	69/79 (87; 78–93)	5 (2–21)	6: 65	1.36 (0.58–15.57)	3 (1–31)	56/66 (85; 73–92)	5 (2–21)	5: 70	1.235 (0.58–6.93)	3 (1–24)	13/13 (100; 72–100)	6 (3–12)	8: 46	1.71 (0.64–15.57)	1 (1–31)	0.200	0.397
CRP >5 mg/L	77/78 (99; 92–100)	5 (1–12)	17: 75	96 (13–436)	7 (1–63)	65/66 (98; 91–100)	4 (1–11)	17: 77	96 (13–394)	7 (1–20)	12/12 (100; 70–100)	5.5 (3–12)	17: 67	99 (33–436)	9 (1–63)	1	1
PCT >0.5 μg/L	52/57 (91; 80–97)	6 (2–13)	4: 63	1.87 (0.57–22.99)	3 (1–11)	42/47 (89; 76–96)	5 (2–11)	2: 69	1.735 (0.57–9.92)	3 (1–9)	10/10 (100; 66–100)	7.5 (4–13)	10: 40	3.475 (0.8–22.99)	4.5 (1–11)	0.574	0.739
PT >1	19/53 (36; 23–50)	6 (3–26)	0: 26	1.11 (1.01–1.87)	1 (1–6)	17/45 (38; 24–53)	6 (3–19)	0: 24	1.11 (1.04–1.87)	1 (1–6)	2/8 (25; 4–64)	16 (6–26)	0: 50	1.085 (1.01–1.16)	1 (1–1)	0.696	0.847
aPTT >36 s	21/37 (57; 40–72)	6 (2–32)	0: 24	41 (36.3–122.4)	6 (2–13)	14/29 (48; 30–67)	6 (2–9)	0: 36	39.8 (36.3–69.6)	2 (1–10)	7/8 (88; 47–99)	8 (6–32)	0: 0	66.3 (37.4–122.4)	4 (1–13)	0.104	0.264

Data are median (range) or frequencies (percentage; 95% confidence interval).

(Adjusted) *P* values refer to the difference between PUUV and DOBV groups in "Proportion of patients with abnormal laboratory test results".

Onset and duration of symptom (Me; Min–Max) are calculated only for patients with abnormal laboratory findings.

Duration is defined as the number of subsequent days with abnormal laboratory findings from onset. Not all laboratory measurements were taken each day, therefore the medical expert (EP) imputed unambiguous days with abnormal laboratory findings for the hospitalized patients.

* Proportion (%) of patients with the onset of individual abnormal laboratory findings.

All measurements from patients are taken into account (including measurements after day 25 of illness).

eGFR–glomerular filtration rate estimated using the Cockcroft–Gault equation (mL/min/1.73 m^2^); ALT–alanine aminotransferase; CRP–C reactive protein; PCT–procalcitonin; PT–Prothrombin time (expressed as international normalized ratio); aPTT–activated partial thromboplastin time

### Symptoms and signs

The most regular and usually the initial clinical sign was fever, which was present in 86% of patients and lasted for 4 (1–9) days. The median highest temperature was 39.3°C (38°C–41°C). Headache was reported by 65% of patients, mostly appearing early in the course of illness and with a median duration of 4 days. The other early symptom was myalgia, present in 40% of patients, most prominent on days 3 and 4, and lasting for a median of 5.5 days.

Oliguria/anuria was present on day 6 (4–10) of illness and lasted for 2 (1–12) days; it was observed in 28% of patients. Diuresis increased afterwards, with polyuria (≥2500 mL/day) that typically appeared on day 9 of illness and persisted for 7 (1–37) days; the median of the most abnormal values was 6600 (2500–12000) mL. Among the 81 patients, 10 (12%) required dialysis that started on day 9 (3–21) of illness and lasted for 3.5 (1–28) days.

Sinus bradycardia was detected in 35% of the HFRS patients; it appeared on day 9.5 (4–26) of illness and was mostly of short duration. Thirty-five percent of HFRS patients reported acute transient myopia; it appeared on day 5 (2–12) of illness and lasted for 2 (1–33) days. Other signs and symptoms shown in Table 2 were detected or reported in ≤30% of patients with HFRS.

Comparison of HFRS caused by PUUV or DOBV in our patient sample found that the proportions of patients with headache and myalgia were higher in those infected with PUUV, whereas the proportions of patients with ascites, pleural effusion, bleeding, oliguria/anuria, and those who needed dialysis were higher in the group infected with DOBV (S2 Fig). However, not all the differences were statistically significant (Table 2).

### Laboratory parameters

Leukocytosis was identified in 55% of patients and thrombocytopenia (<130 x 10^9^/L) in nearly all (95%) patients on hospital admission. The lowest platelet count was observed on day 5 of illness (S3 Fig) and rose to normal values in a median of 4 days.

Elevated levels of CRP (>5 mg/L) and PCT (>0.5 μg/L) were present in 99% and 94% of patients, respectively, and were detected on median day 5 of disease; i.e., on admission to hospital. In 64/81 (79%) patients the level of CRP was ≥50 mg/L. Elevated creatinine was found in 94% of patients at 1–12 (median 6) days after the onset of illness. The majority of patients were discharged from hospital with some degree of renal failure.

Elevated ALT values (usually 2–3 x upper normal values) were found in 53% of patients; aPTT was prolonged in 21/37 (57%) patients (Table 3).

Comparison of HFRS caused by PUUV or DOBV in our sample showed several differences. Patients with DOBV infection had more pronounced leukocytosis and higher levels of PCT, as well as higher proportions with elevated ALT values and prolonged aPTT.

These findings and several others are shown in Table 3 and S3 Fig.

## Discussion

Our study provides detailed information on the onset, duration, and magnitude of clinical signs/symptoms and laboratory abnormalities in HFRS, together with information on the proportion of Slovenian patients with individual abnormalities present during the course of illness. The study corroborates previous findings on the course and outcome of HFRS, including the frequency of the main clinical signs/symptoms and laboratory abnormalities [5, 6, 9, 19–23] and the existence of PUUV and DOBV as causative agents of the disease in Slovenia [5, 6]; it also provides further information on differences in the corresponding disease severity [5, 6]. A more severe clinical course in patients infected with DOBV was suggested by the higher proportion of patients fulfilling the criteria for severe disease, such as the presence of oliguria/anuria and the need for dialysis; higher proportions of patients with ascites, pleural effusions, and major bleeding; and longer hospitalization. Although not all the differences were statistically significant they showed identical tendencies (Table 2).

Our study confirms that middle-aged men pursuing outdoor activities during the summer are the key affected population, and also that the disease is seldom recognized by primary care physicians. Although the presence of HFRS in Slovenia has been known for more than 35 years [24, 25], and despite heightened medical and public (media) interest during a large epidemic in 2012 [14, 26], only 4/81 (5%) patients in our study were referred to hospital with a suggestion of having HFRS (Table 1). One of the difficulties of diagnosis relates to the unusual laboratory findings; namely, HFRS, although a viral disease, is characterized by laboratory parameters suggesting bacterial infection [5, 27, 28]. Our study found that CRP and PCT levels were elevated in >90% of patients, that elevations were typically present on admission to hospital, and that their median duration was 7 and 3 days, respectively (Table 3).

It is well accepted that the main clinical hallmarks of HFRS are renal failure and hemorrhagic manifestations, and that the disease is characterized by laboratory findings indicating renal function impairment, thrombocytopenia, and elevated ALT levels. Indeed, thrombocytopenia, some degree of renal function impairment, and elevated ALT levels were observed in almost all our patients. Thrombocytopenia was usually present on admission (5 days after onset of illness) and lasted for 4 (1–16) days. The lowest platelet count (median 55 x 10^9^/L) was on day 5 of illness, at the end of fever; thereafter, no further decline in platelet count occurred. In contrast to the laboratory findings, only 28% of our patients had clinically overt renal failure manifested as oliguria or anuria (21% with PUUV, 67% with DOBV). This finding is in accordance with several [5, 6, 29] but not all previous reports; for example, in a German study oliguria was described in as many as 32/75 (43%) patients with PUUV infection [20]. Nevertheless, in the present study the frequency of oliguria was even lower than the occurrence of blurred vision, which is the result of transient myopic shift due to lens thickening, and is considered a pathognomonic sign of HFRS. The reported rates of blurred vision vary considerably: from 22% to 87% in patients with PUUV infection [5, 6, 9, 30, 31]. In our study, acute myopia was reported by 35% of patients. It appeared 2–12 (median 5) days after the onset of illness and was of short duration (median 2 days). Similar findings were obtained for sinus bradycardia, another striking but rarely reported transient manifestation of HFRS [5, 32] that is usually associated with hypotension [33]. In the present study it was detected in 28/81 (35%) patients, but started later in the course of illness than blurred vision and was mostly of short duration (Table 2).

Clinically overt bleeding is rare in European patients with HFRS [5, 6, 9]. In our study it was present in only 7/81 patients (9%), which is low for a disease entitled “hemorrhagic” and further supports the suggestion that some other designation, such as hantavirus disease, would be more appropriate.

There is increasing evidence of similarities between HFRS and (cardio)pulmonary syndrome, as pulmonary involvement can also be present in HFRS [34]. In a German report, abnormal chest X-ray findings suggesting pulmonary infiltrates or pleural effusions were present in 12/75 (16%) of patients with PUUV infection [20]. In the present study, minor alveolar pulmonary infiltrates were detected in only 2/66 (3%) patients with PUUV infection and 2/15 (13%) with DOBV. Pleural effusions were present in 12/81 (15%) patients: in 5/66 (8%) with PUUV infection and in 7/15 (47%) with DOBV (*P* = .012). The effusions were detected on median day 8 of illness and were often associated with the presence of ascites (Table 2), suggesting vascular leakage and/or over-hydration.

The design of our study, which is–to the best of our knowledge–the first comprehensive prospective study presenting the daily evolution of clinical and laboratory findings in HFRS, has enabled assessment of the proportion of patients with individual signs/symptoms/laboratory abnormalities present on each day of illness (S2 and S3 Figs). The study also allowed evaluation of the onset, duration, and magnitude of several clinical signs/symptoms and laboratory abnormalities (Tables 2 and 3). Our results showed that the classic evolution through five distinct phases (febrile, hypotensive, oliguric, polyuric, and convalescent) is often unclear.

Not all patients with HFRS but only those hospitalized in a subset of Slovenian hospitals and for whom serial clinical findings and laboratory parameters were available were enrolled in the present study, which might represent selection bias. The other main limitations of the study are the small number of patients with DOBV infection and the incomplete capture of some laboratory and clinical data (collection errors), resulting in serial evaluation of individual parameters in fewer patients. In addition, since chest X-ray and abdominal ultrasound were not performed daily, we possibly missed some information on the frequency, onset, and duration of pulmonary infiltrates and pleural/abdominal effusions. Furthermore, in contrast to symptoms, some HFRS signs and nearly all laboratory parameters could be (reliably) evaluated only during hospitalization (the median time of illness before hospitalization was 5 days). Thus, the actual onsets of signs and laboratory parameters were most likely to be earlier than recorded in our study; consequently, the durations of abnormalities could be longer if monitored systematically from the very beginning of the disease.

### Conclusions

Knowledge of the exact onset, duration, and magnitude of clinical signs/symptoms and laboratory abnormalities in HFRS, as well as information on the proportion of patients with individual abnormalities during the course of illness, are important in management of HFRS. The classic evolution of HFRS through five distinct phases (febrile, hypotensive, oliguric, polyuric, and convalescent) is often not evident.

## Supporting information

S1 Fig Timeline dynamics of specific IgG and IgM antibodies, and plasma viral load in patients infected with Puumala or Dobrava virus. (PDF)

S2 Fig Sequential evolution of clinical parameters in patients having hemorrhagic fever with renal syndrome caused by Puumala (PUUV) or Dobrava virus. (PDF)

S3 Fig Sequential evaluation of laboratory parameters in patients having hemorrhagic fever with renal syndrome caused by Puumala (PUUV) or Dobrava virus. (PDF)
