# Supplementary material for: Sequential assessment of clinical and laboratory parameters in patients with hemorrhagic fever with renal syndrome
Source: PLoS One. 2018 May 23;13(5):e0197661. doi: 10.1371/journal.pone.0197661 (PMC5965875; doi:10.1371/journal.pone.0197661)
Supplement: S1 Fig — (PDF) [file pone.0197661.s001.pdf]

Supplementary figure 1. Timeline dynamics of specific IgG and IgM antibodies, and plasma viral load in patients infected with Puumala or Dobrava virus.

The timeline was constructed from samples from 18 patients with PUUV and 11 with DOBV. Average values across days of illness are connected with a solid line. In order to exclude bias, calculations were made for measurements until day 25 of illness, for patients in whom at least four measurements were obtained. Parameter range (0 to 90th percentile of all values) is indicated by dotted lines. A cut-off value for IgM is R > 1 and for IgG is R > 1.2. The average value measured at admission to the hospital was 2.8 (max 8.8) for IgM and 2.3 (max 5.0) for IgG. The viral load ranged from $6.8x10^{1}$ RNA copies/ml to $2.5x10^{7}$ RNA copies/ml for patients infected with DOBV, and from $1.0x10^{1}$ RNA copies/ml to $9.1x10^{6}$ RNA copies/ml for patients infected with PUUV. Light shaded area corresponds to days of illness when up to 50% of patients were still hospitalized. Dark shaded area corresponds to days of illness when up to 33% of patients were still hospitalized.

PUUV, Puumala virus; DOBV, Dobrava virus

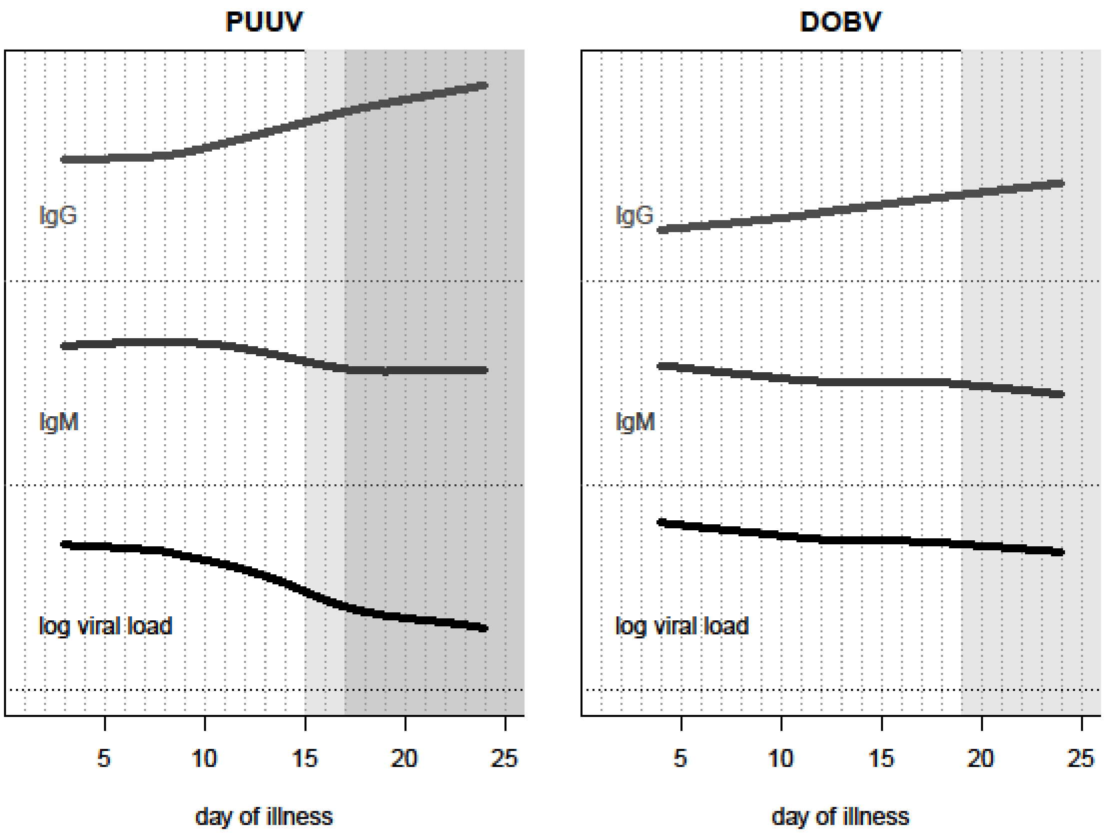
PUUV
DOBV
IgG
IgM
log viral load
5
10
15
20
25
day of illness
