# Supplementary material for: Sequential assessment of clinical and laboratory parameters in patients with hemorrhagic fever with renal syndrome
Source: PLoS One. 2018 May 23;13(5):e0197661. doi: 10.1371/journal.pone.0197661 (PMC5965875; doi:10.1371/journal.pone.0197661)

Supplementary figure 2. Sequential evolution of clinical parameters in patients having hemorrhagic fever with renal syndrome caused by Puumala (PUUV) or Dobrava virus (DOBV)

Boxplots are plot for each day of illness (black dots correspond to data points more than 1.5 IQR from 1st or 3rd quartile). Thin lines connect measurements of the same patient. Red lines in the graphs are for “normal boundaries” for each individual clinical variable. Black part of barplot represents the proportion (%) of patients having a certain clinical sign/symptom during time course. PUUV infected group of patients in the left and DOBV in the right part of the picture.

There are 81 patients in the data set. Number of patients with available values for each variable is displayed in the title.

a) body temperature (°C). Normal boundary is set to 38. There are 72 patients with values for this variable.

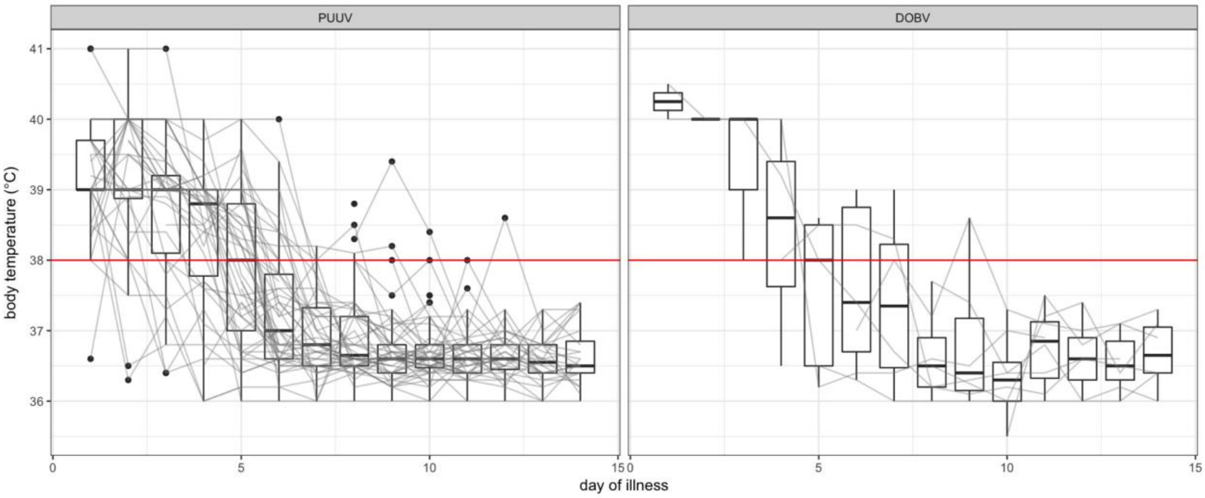

b) systolic blood pressure (mmHg). Normal boundaries are set to 90 and 140. There are 73 patients with values for this variable.

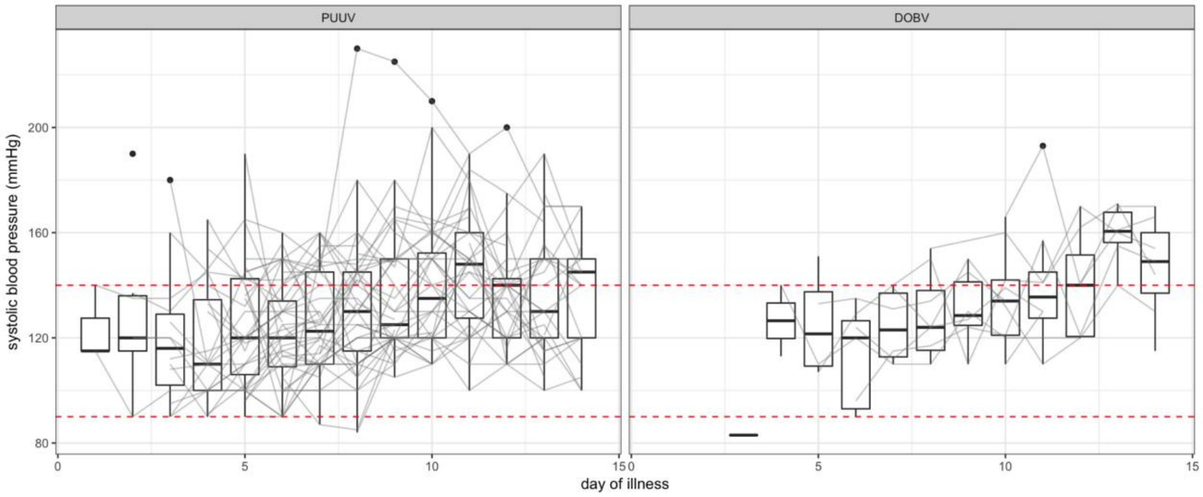

c) diuresis (mL/day). Normal boundaries are set to 500 and 2500. There are 64 patients with values for this variable.

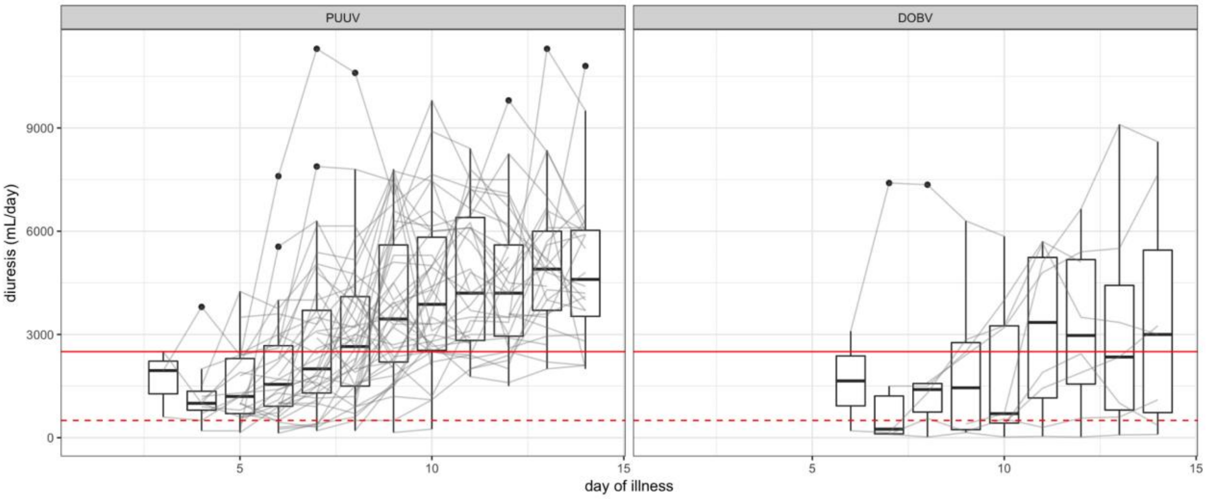

d) headache. There are 81 patients with values for this variable.

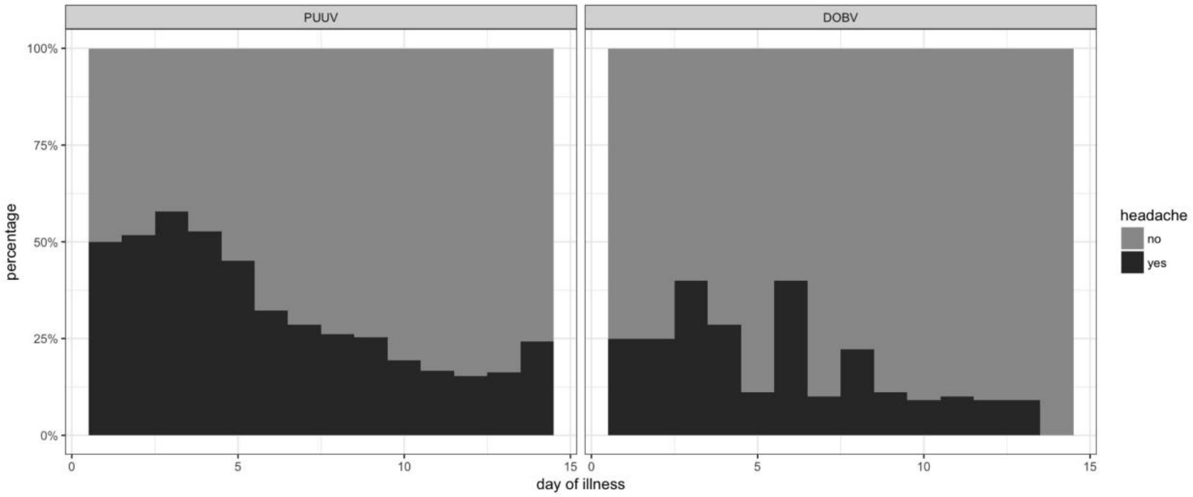

e) myalgia. There are 81 patients with values for this variable.

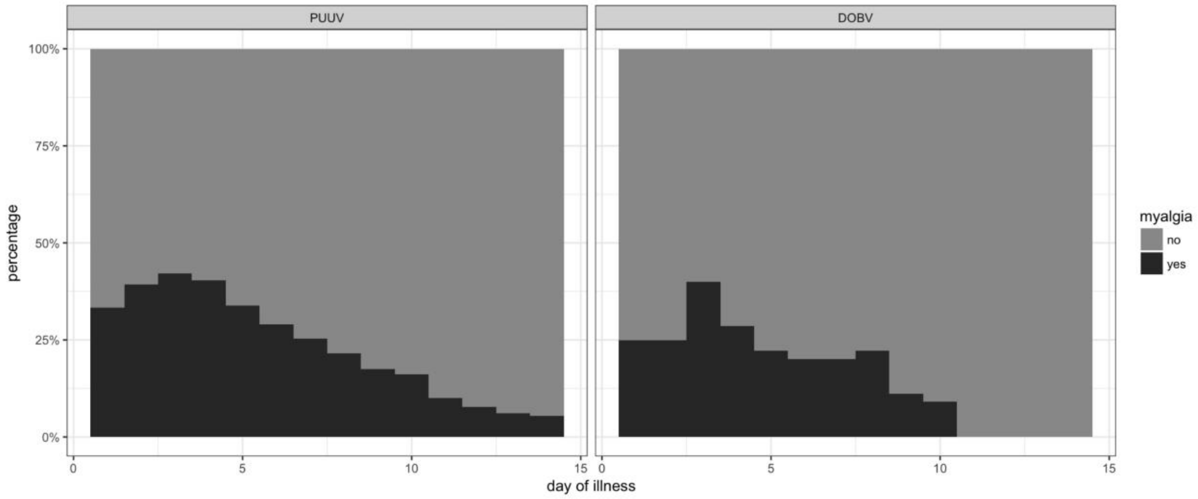

f) dizziness. There are 81 patients with values for this variable.

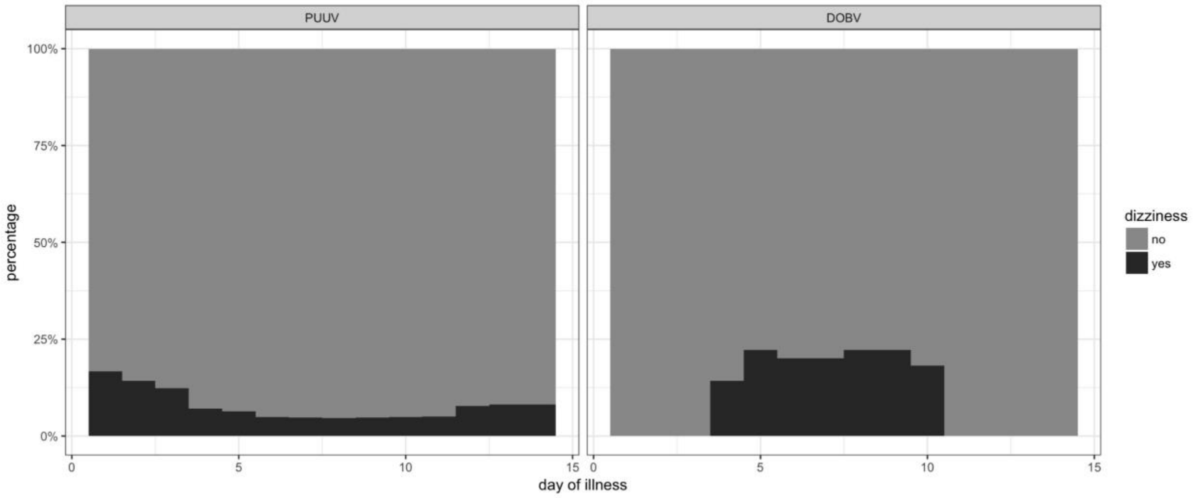

g) acute myopia. There are 81 patients with values for this variable.

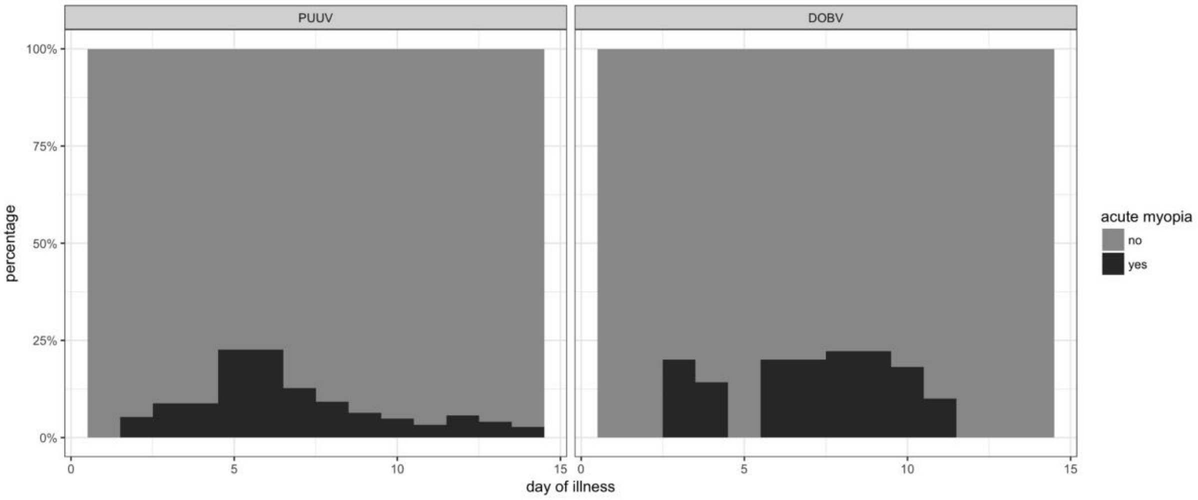

h) insomnia. There are 81 patients with values for this variable.

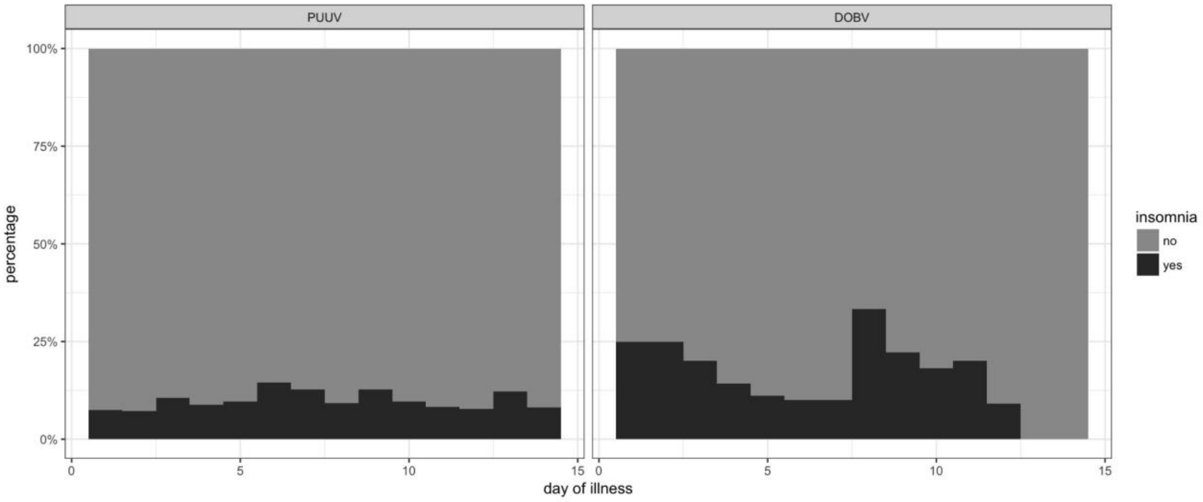

i) bradycardia. There are 81 patients with values for this variable.

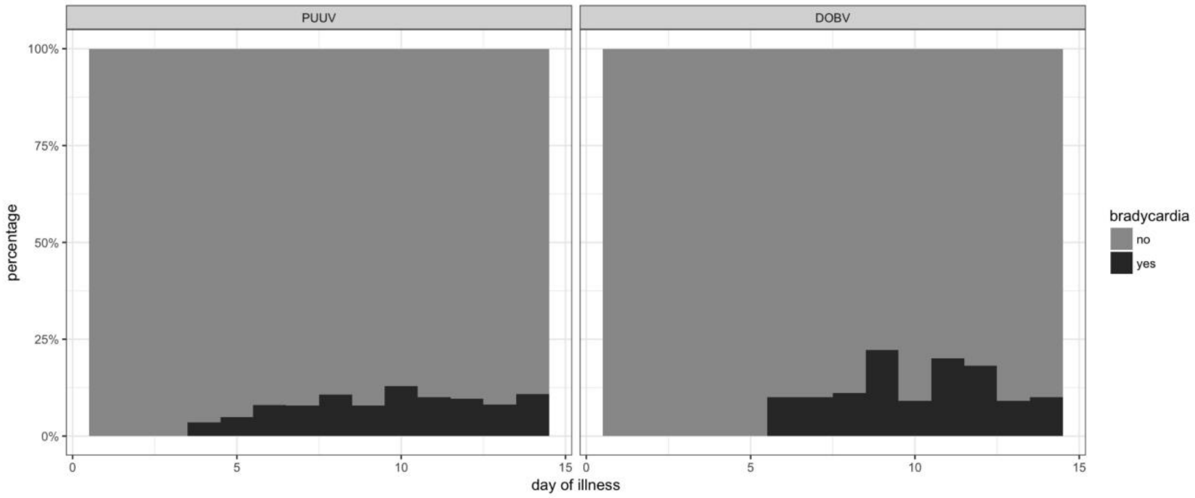

j) ascites. There are 81 patients with values for this variable.

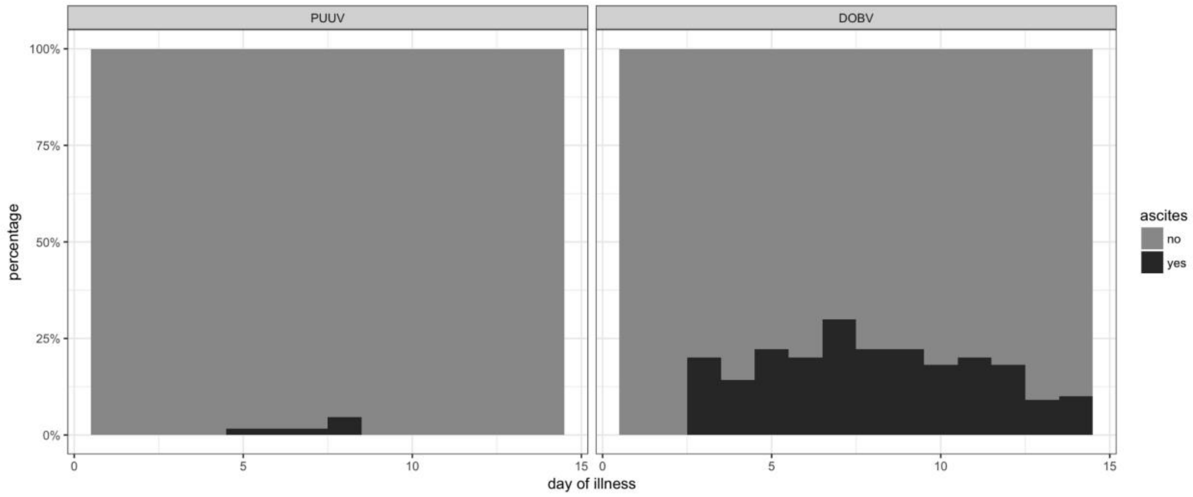

k) pleural effusion. There are 81 patients with values for this variable.

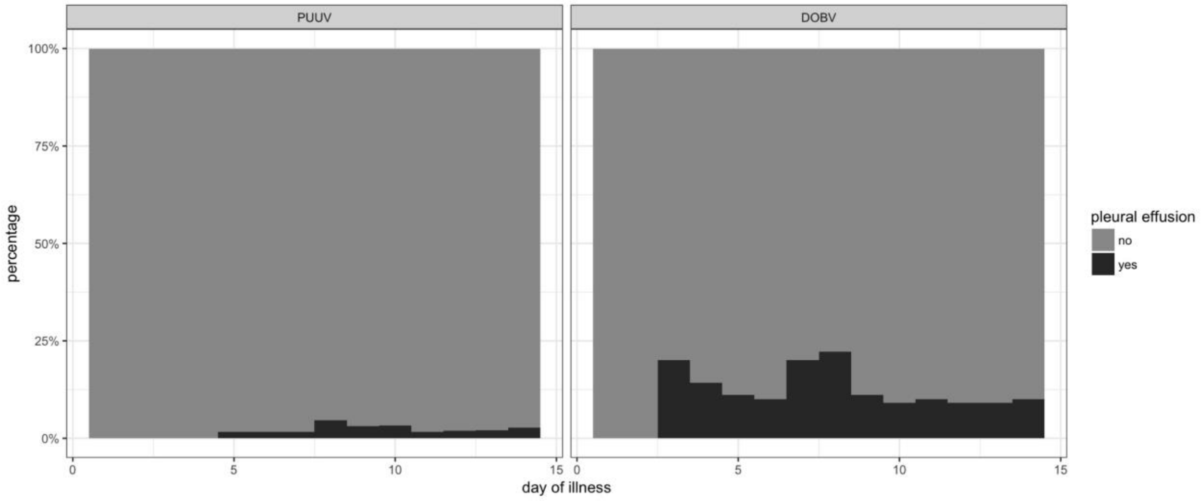

l) bleeding major (from gastrointestinal, genitourinary and pulmonary sites). There are 81 patients with values for this variable.

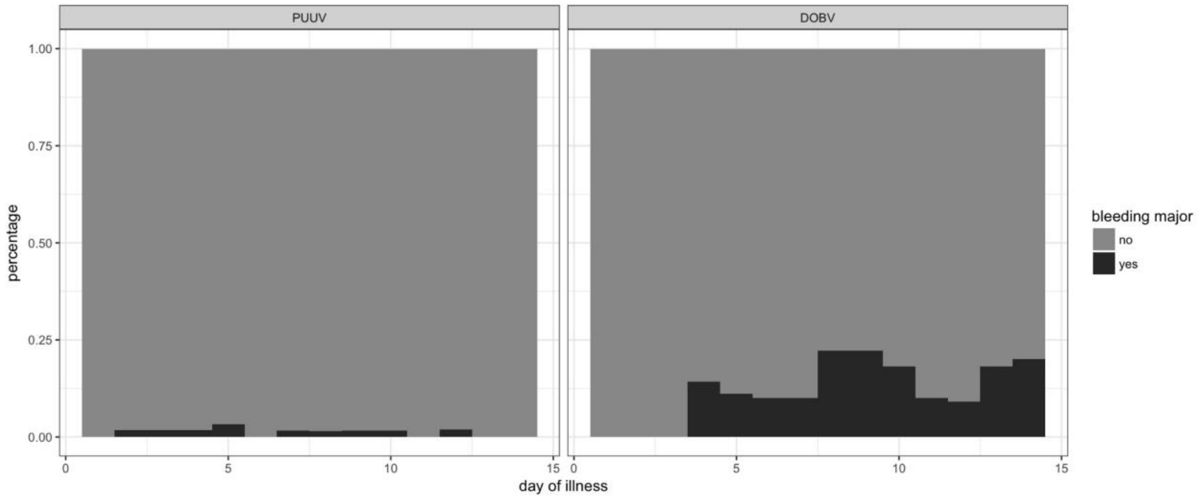

m) bleeding only minor (petechiae, ecchymosis, epistaxis). There are 81 patients with values for this variable.

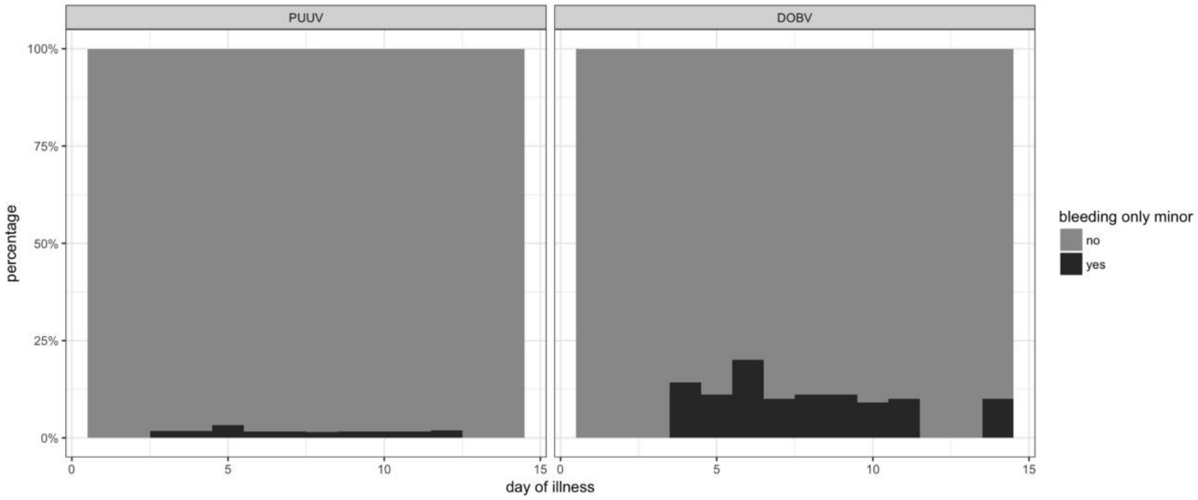

Supplement: S2 Fig — (PDF) [file pone.0197661.s002.pdf]
