# Supplementary material for: Sequential assessment of clinical and laboratory parameters in patients with hemorrhagic fever with renal syndrome
Source: PLoS One. 2018 May 23;13(5):e0197661. doi: 10.1371/journal.pone.0197661 (PMC5965875; doi:10.1371/journal.pone.0197661)

Supplementary figure 3. Sequential evaluation of laboratory parameters in patients having hemorrhagic fever with renal syndrome caused by Puumala (PUUV) or Dobrava virus (DOBV)

Measured in serum. Boxplots are plot for each day of illness (black dots correspond to data points more than 1.5 IQR from 1st or 3rd quartile). Thin lines connect measurements of the same patient. Red lines in the graphs denote “normal boundaries” for every laboratory variable. PUUV infected group of patients in the left and DOBV in the right part of the picture.

There are 81 patients in the data set. Number of patients with available values for each variable is displayed in the title.

a) platelet count (10^9/L). Normal boundaries are set to 130 and 400. There are 81 patients with values for this variable.

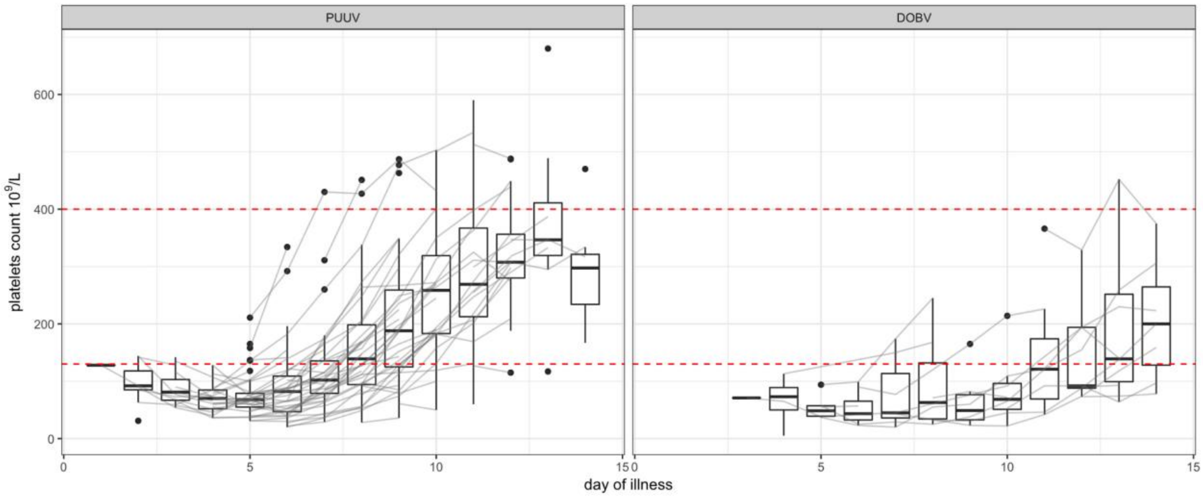

b) leucocyte count (10^9/L). Normal boundaries are set to 4 and 10. There are 78 patients with values for this variable.

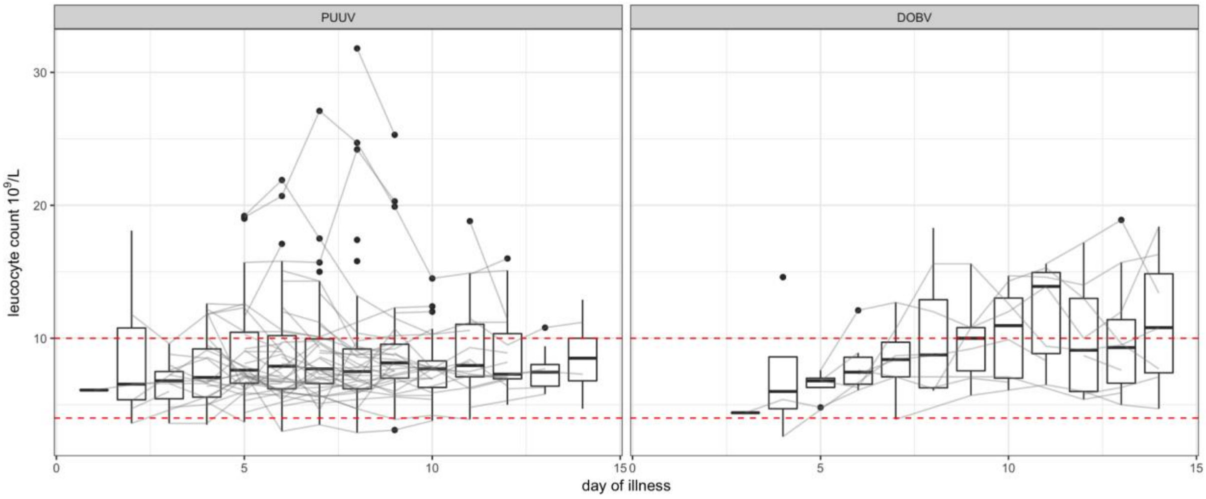

c) creatinine (μmol/L). There are 80 patients with values for this variable. Normal boundaries are set to 44 and 97.

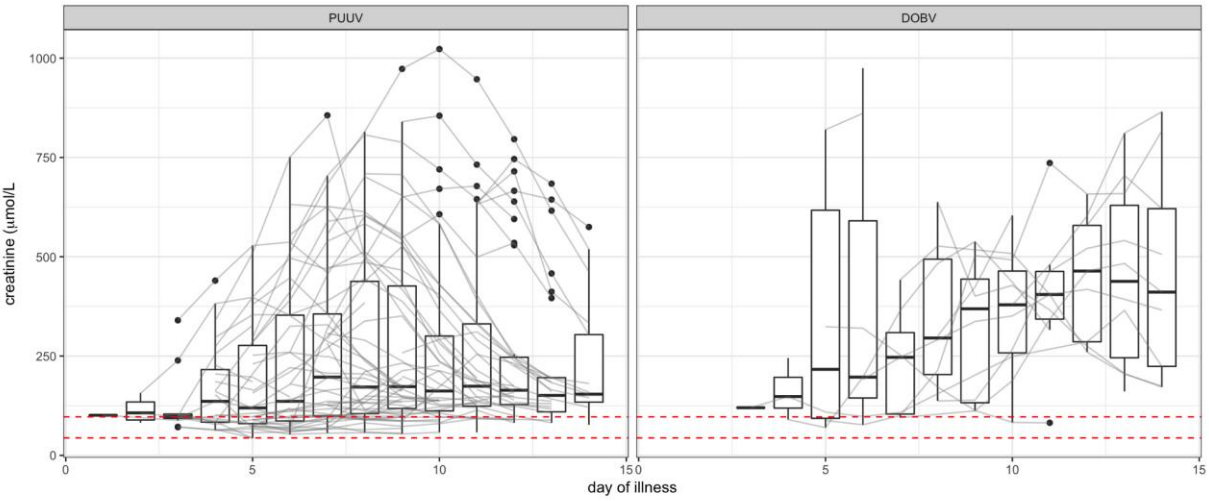

d) estimated glomerular filtration rate (eGFR, mL/min/1.73 m^2). Normal boundary is set to 60. There are 77 patients with values for this variable.

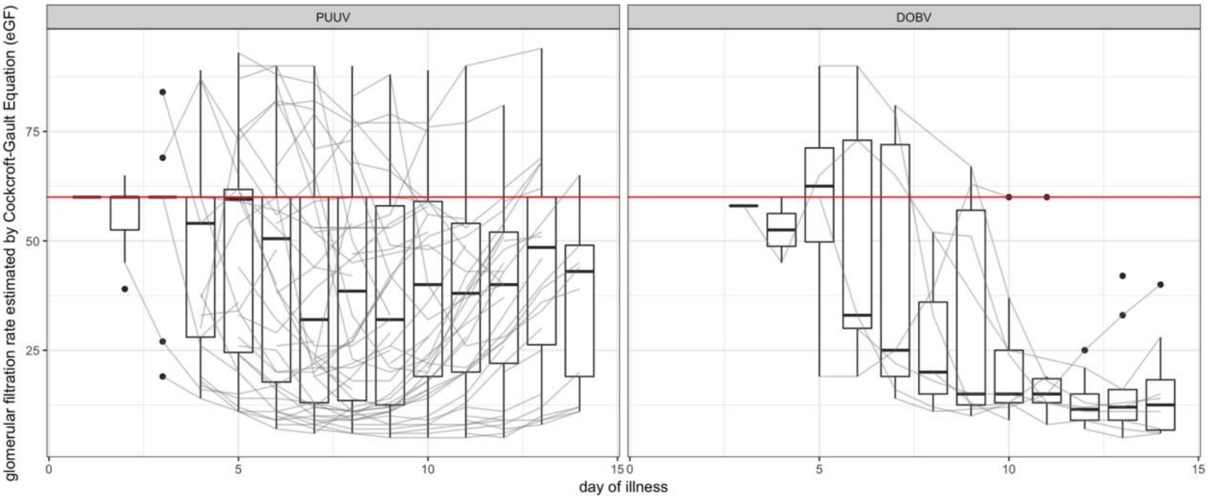

e) alanin–aminotransferase (ALT, μkat/L). Normal boundary is set to 0.56. There are 79 patients with values for this variable.

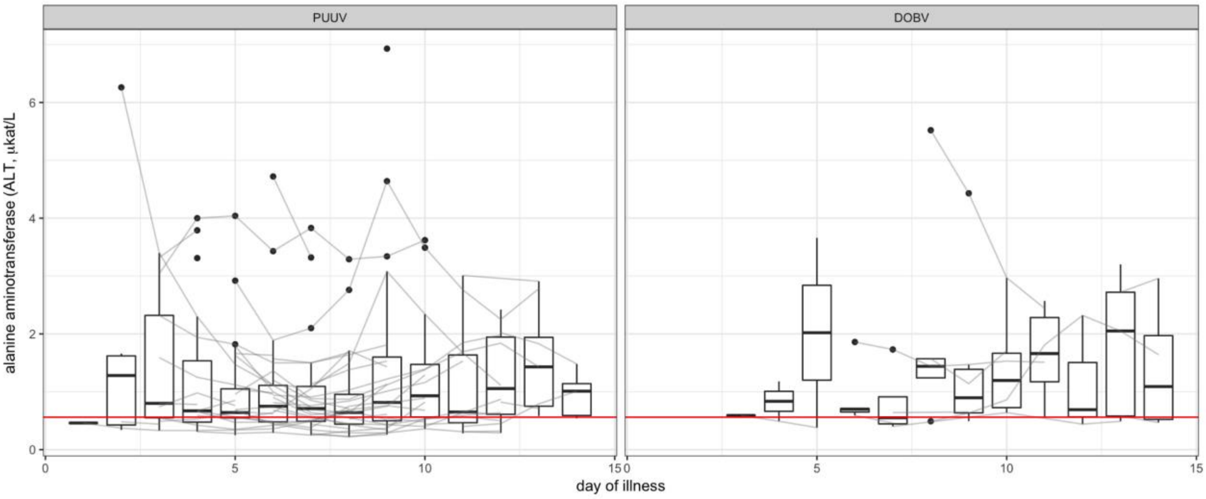

f) C-reactive protein (CRP, mg/L). Normal boundary is set to 5. There are 78 patients with values for this variable.

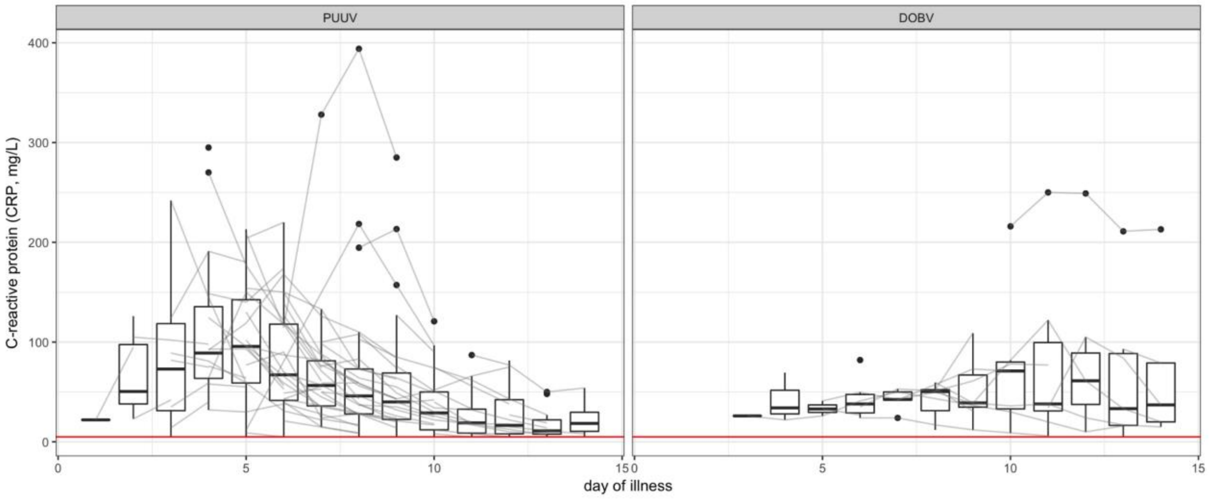

g) procalcitonine (PCT, μg/L). Normal boundary is set to 0.5. There are 57 patients with values for this variable.

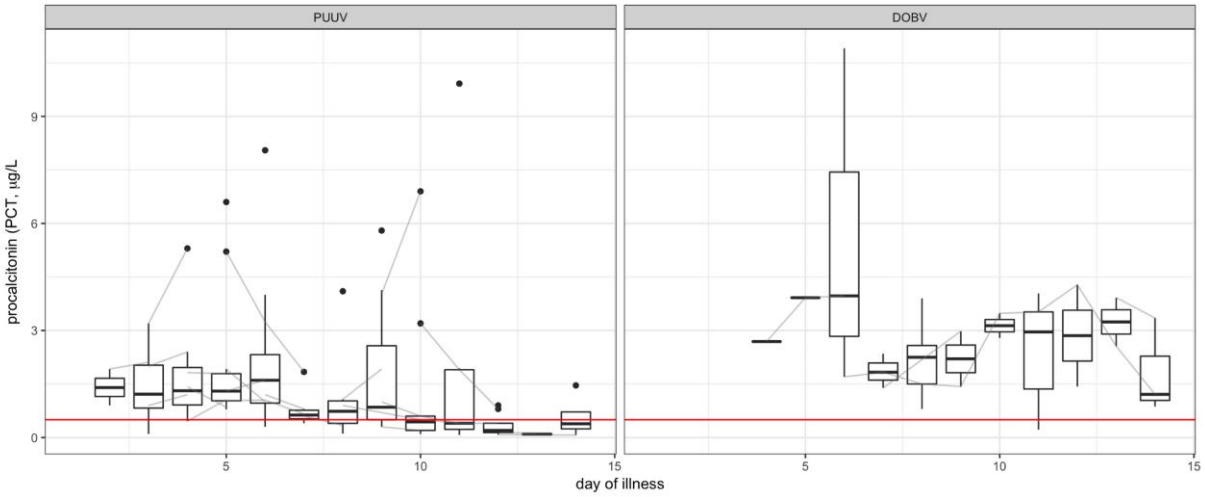

h) prothrombin time (PT, INR). Normal boundaries are set to 0.7 and 1. There are 52 patients with values for this variable.

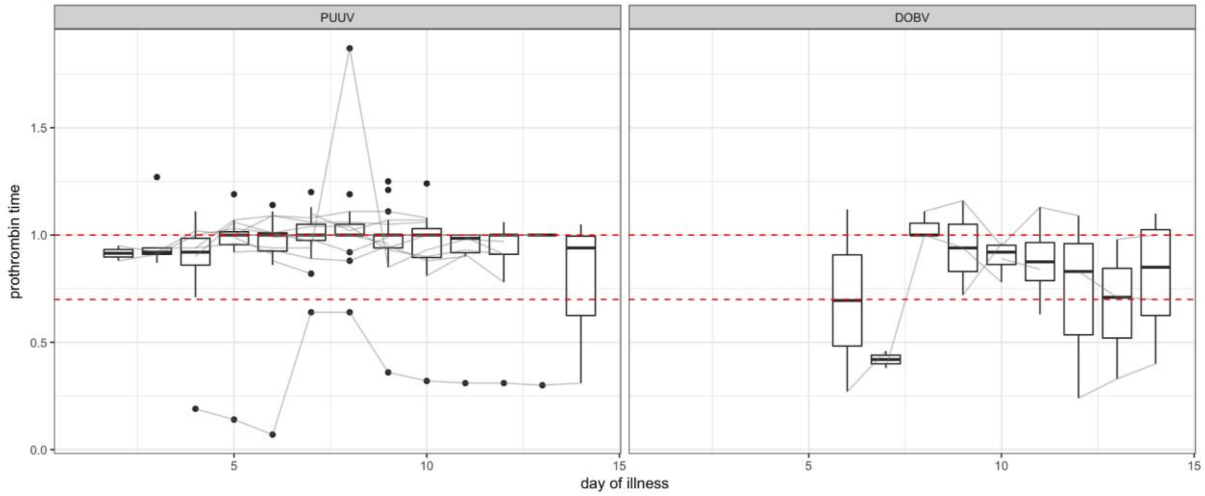

i) activated partial thromboplastine time (APTT, s). Normal boundaries are set to 23 and 36.

There are 36 patients with values for this variable.

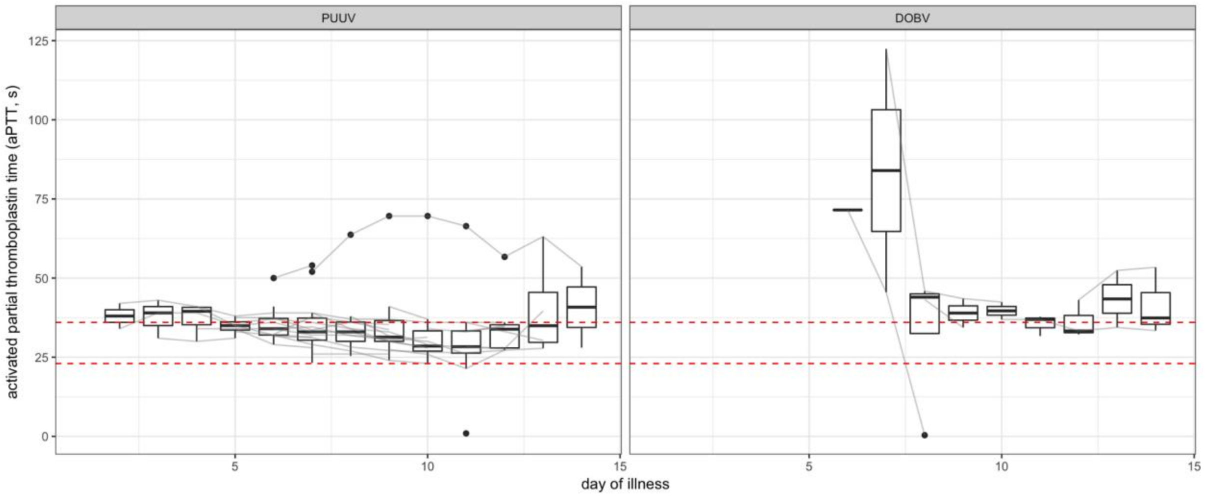

Supplement: S3 Fig — (PDF) [file pone.0197661.s003.pdf]
